# Metabolomic Profiling Has the Potential to Differentiate Between Iron Deficiency Anemia and Anemia of Inflammation

**DOI:** 10.3390/metabo16070498

**Published:** 2026-07-15

**Authors:** Triin Paabo, Eliis Grigor, Egon Taalberg, Natalja Luppova, Eliise-Rosalinda Raudmäe, Piret Mihkelson, Alan Altraja, Ain Kaare, Rando Porosk, Kalle Kilk

**Affiliations:** 1 Department of Hematology and Bone Marrow Transplant, Tartu University Hospital, 50406 Tartu, Estonia; 2 Department of Biochemistry, Institute of Biomedicine and Translational Medicine, University of Tartu, 50411 Tartu, Estonia; 3 Hematology Centre, North Estonia Medical Centre, 13419 Tallinn, Estonia; 4 United Laboratories, Tartu University Hospital, 50406 Tartu, Estonia; 5 Lung Clinic, Tartu University Hospital, 50406 Tartu, Estonia; 6 Department of Pulmonology, Institute of Clinical Medicine, Faculty of Medicine, University of Tartu, 50406 Tartu, Estonia

**Keywords:** metabolomics, iron deficiency, anemia of inflammation, biomarkers

## Abstract

**Highlights:**

**What are the main findings?**

- Anemia is associated with metabolomic alterations of polyunsaturated fatty acid metabolism.
- Metabolomic profiling could distinguish anemia subtypes.

**What are the implications of the main findings?**

- The preliminary results need validation in larger cohorts, but metabolomics may contribute to the classification of anemia subtypes not adequately distinguished by existing biomarkers.

**Abstract:**

Background/Objectives: Discrimination between iron deficiency anemia (IDA) and anemia of inflammation (AI), the major causes of anemia, remains a challenge and novel biomarkers are needed. This prospective study characterizes metabolomic changes in patients with anemia, compares IDA and AI, and evaluates the ability of metabolomic profiling to categorize otherwise unclassifiable anemia (UA) cases. Methods: In the single-center cross-sectional study, patients with anemia were classified as IDA, AI, or their combination (IDA + AI) according to traditional iron biomarkers. Targeted metabolomic analysis was conducted with tandem mass spectrometry using the MxP^®^ Quant 500 kit, with 617 individual metabolites and 227 calculated parameters in the final analysis. Results: The final cohort included 70 patients with anemia and 27 controls. The concentrations of six metabolites and six calculated parameters were significantly different in patients with anemia compared to controls. Anemia was associated with decreased concentrations of several polyunsaturated fatty acid (PUFA)-containing triglycerides (*n* = 3) and phosphatidylcholines (*n* = 2), as well as with increased asparagine/aspartate and docosahexaenoic acid/eicosapentaenoic acid ratio. Multivariate analysis using only metabolomic data differentiated between IDA and AI with a general classification accuracy of 88%. Unclassifiable anemia samples were reclassified mostly as IDA (*n* = 11) or IDA + AI (*n* = 6), with one sample reclassified as AI. Conclusions: Metabolomic markers of PUFAs differ between patients with anemia and controls, indicating possible alterations in lipid metabolism. Our preliminary findings suggest that metabolomic data may be capable of distinguishing between IDA and AI. However, owing to several limitations of the current study, the potential utility of metabolome-based approaches should be confirmed in independent validation studies.

## 1. Introduction

Anemia is estimated to affect 24% of the world’s population, corresponding to 1.9 billion cases [1]. While dietary iron deficiency (ID) is the most common cause of anemia worldwide [1], anemia of inflammation (AI) is the second leading factor in the Western world and the most prevalent cause in hospitalized and chronically ill patients [1,2]. AI is often combined with absolute or functional ID. Despite the expanded knowledge of AI and ID, discrimination remains a clinical challenge and novel biomarkers are needed [2].

Metabolites reflect the current biochemical state of a biological sample and can therefore provide valuable insight into disease-related processes, making them promising biomarker candidates. Metabolite concentrations are highly dependent on nutritional status and are also influenced by genetic regulation and other factors. Hence, anemias due to dietary, genetic or inflammatory causes could all have unique fingerprints in the metabolome. Extensive metabolomic studies have led to the discovery of several new biomarkers and treatment targets of different cancer types and cardiovascular, endocrine, and neurological diseases [3,4,5].

Studies in rats and rhesus monkeys have provided the first insights into possible metabolic fingerprints of ID and iron deficiency anemia (IDA). Gestational and lactational ID has been shown to alter creatinine, glycose, glutamine, glutamate, N-acetylaspartate, myoinositol, and phosphorylcholine metabolism in rats [6]. Other studies in rats have associated reduced iron intake with altered glycerophospholipid, sphingolipid, and fatty acid metabolism [7,8]; lower activities of glycose-6-phosphate dehydrogenase and malic enzyme have also been described [8]. Studies in infant monkeys have connected ID and IDA to a hypometabolic state, altered liver function, differential essential fatty acid production, irregular nucleoside metabolism, and atypical bile acid production [9,10].

A metabolomic study in human infants with IDA revealed decreased concentrations of succinate and several amino acids and increased concentration of inosine in boys and decreased concentration of fumarate in girls [11]. A study of untargeted serum metabolomics analyzing 119 metabolites revealed the altered level of 12 metabolites in IDA patients compared to healthy controls [12]. Metabolomics has been applied to find diagnostic and prognostic markers in autoimmune hemolytic anemia (AIHA) [13], paroxysmal nocturnal hemoglobinuria [14], sickle cell disease [15], hereditary spherocytosis (HS) [16], pyruvate kinase deficiency (PKD) [17], and Diamond-Blackfan anemia [18]. Most of the studies have used healthy controls without anemia as the comparison group, giving a detailed overview of metabolic changes in certain diseases.

In clinical settings, finding the cause of anemia is often the principal question to enable causal treatment. Using healthy people as controls may limit the applicability of the results in the search for novel diagnostic biomarkers for differential diagnosis. In addition, the metabolic consequences of anemia and of tissue hypoxia itself, irrespective of the cause, have not been unequivocally determined. Improved biomarkers for detecting iron deficiency are required, particularly among individuals with inflammatory diseases and organ failure.

We hypothesize that the metabolomic profile differs in patients with anemia compared to controls, distinguishes different anemia subtypes and can determine the cause of anemia in unclassifiable cases. Here we describe the metabolomic changes in anemia, compare the metabolic profile of patients with IDA, AI or their combination (IDA + AI), and test whether metabolomic profiling can help to reclassify patients with unknown anemia.

## 2. Materials and Methods

### 2.1. Study Design and Participants

The study was a prospective, single-center cross-sectional study conducted at Tartu University Hospital from March 2018 to January 2023. Samples of anemic patients were collected from outpatients in the Departments of Hematology and Internal Medicine. The study included patients referred to hematologists or internists due to iron deficiency anemia, anemia of unknown cause or anemia due to chronic diseases. Control individuals were recruited from the outpatient clinics of the Departments of Hematology and Pulmonology and from the general population. The control individuals had to be without anemia at the time of sample collection. Individuals with known pregnancy, hemolytic anemia, or known active malignancies, or those who had received red blood cell transfusion or erythropoietin (EPO) therapy within the last 3 months were not included in the study. Restricting the inclusion site to an outpatient setting (i.e., participants did not have any acute illnesses requiring hospitalization) was done to minimize heterogeneity and avoid bias due to deteriorated general condition. The presence of chronic diseases and/or medicine (including iron) intake was not an exclusion criterion for the study (neither for patients with anemia nor controls).

All study subjects provided written informed consent and filled in the study questionnaire. The data obtained from study questionnaires and from electronic patient journals included age, sex, height, weight, body mass index (BMI), and concomitant chronic diseases. Blood samples for hematology and clinical chemistry analysis were collected as part of routine clinical practice. Two additional serum tubes were collected for metabolomic analyses.

### 2.2. Classification of Anemia

Patients with peripheral blood hemoglobin (Hb) concentration below the lower limit of the national reference value (121–150 g/L for women, 134–170 g/L for men) [19] were classified as having anemia. Hb concentration within the reference limits and ferritin below the upper normal limit of the reference value (ferritin < 150 μg/L for women, <400 μg/L for men) was required to be included in the control group. Subjects with anemia were classified into four groups (IDA, AI, IDA + AI, unclassifiable anemia (UA)) according to ferritin, transferrin saturation (TSAT), soluble transferrin saturation/log_10_ferritin (sTfR-F) index, C-reactive protein (CRP), and estimated glomerular filtration rate (eGFR) (Table 1). The applied classification is based on the criteria previously used by van Santen et al. 2011 [20], using a CRP cut-off of 5 mg/L, including eGFR < 60 L/min/1.73 m^2^ to the criteria as a possible factor of anemia associated with renal failure [21], and modifying the sTfR-F index cut-off considering the reference value of the manufacturer (Roche Diagnostics, Rotkreuz, Switzerland).

### 2.3. Experimental Methods

Hematological and clinical biochemistry analyses were performed at the United Laboratories of Tartu University Hospital following accredited protocols and quality standards identical to those in routine clinical practice.

Serum samples were collected in Vacuette^®^ CAT Serum Clot Activator tubes (Greiner Bio-One GmbH, Kremsmünster, Austria). The samples were left to clot at room temperature, then centrifuged at 1500× *g* for 15 min at 15 °C. The obtained serum samples were aliquoted and stored afterwards at −80 °C until further analysis.

Metabolites were measured with the MxP^®^ Quant 500 targeted metabolomics kit (Biocrates Life Sciences AG, Innsbruck, Austria) using a mass spectrometer Xevo TQ-XS (Waters Corporation, Milford, MA, USA). Sample preparation was carried out according to the instructions provided by the manufacturer. The samples were analyzed in random order with at least three replicates of external quality control present in each batch. Batch corrections were done with the quality control sample and confirmed by low coefficient or variation between the control group samples of different batches. Concentrations of metabolites were calculated using the MetIDQ™ software (Biocrates Life Sciences AG). Calculated parameters were the metabolite sums and ratios pre-defined by the MetaboINDICATOR™ add-on tool provided by the manufacturer. The parameters for newborn screening were excluded.

### 2.4. Statistics

Considering the non-normal distribution of the hematological and clinical biochemistry parameters based on the visual estimation of Q-Q plots, results are presented as median values and interquartile range. Chi-squared test for categorical variables and non-parametric tests for continuous variables (Wilcoxon or Kruskal–Wallis test, where appropriate) were used to test the distribution of general characteristics; *p*-values less than 0.05 were considered statistically significant.

Metabolites that had missing values in more than 30% of samples were excluded from the main analyses. The missing values were distributed evenly between the main study groups (chi-square test *p* = 0.1). In total, imputation was used in 0.92% of measured metabolite concentrations; missing values were substituted with a random forest algorithm from the missForest package [22].

For analyzing metabolomic data, log_10_ transformation was used for all metabolites and calculated parameters. In univariate analysis, Student’s t-test was used to compare the concentration of metabolites in the anemia and control groups; the model was adjusted for age and sex. Analysis of variance (ANOVA) with Tukey’s *post hoc* test was performed for subgroup (IDA, AI, IDA + AI, UA) metabolite comparisons; the model was adjusted for age, sex, hemoglobin and creatinine concentration. The effect of including covariates in nested linear models was assessed using differences in Akaike information criterion (ΔAIC). Benjamini–Hochberg procedure was used for false discovery rate (FDR) correction. FDR-corrected *p* < 0.05 was considered statistically significant. Tests with an original *p*-value < 0.05 that lost their significance due to the FDR procedure were considered to have borderline significance. Fold change and Cohen’s d were used as descriptives of effect size.

In multivariate analysis, sparse partial least squares discriminant analysis (sPLS-DA) was performed to test whether only metabolic data discriminates between different participant groups and can reclassify patients with UA. The model was built using samples from IDA and AI patients as training sets and then applied to samples from patients with an IDA + AI combination and with UA. The balance of predictive performance and overfitting was optimized with the tuning function of the MixOmics package and final performance was assessed with 5 folds and 100 repeats. No adjustment or inclusion of clinical parameters was used. The optimal number of parameters was calculated by the tuning function of the MixOmics [23] package. The top ten variables with the highest variable importance in projections (VIP) scores are reported; model performance was assessed by overall and balanced error rate.

Statistical tests were performed with RStudio (version 2024.04.1; Posit Software, PBC, Boston, MA, USA). sPLS-DA, calculation of VIP scores and plotting were performed with the MixOmics [23], gglpot2 [24], and dplyr [25] packages.

## 3. Results

### 3.1. Characteristics of Participants

Samples were collected from 112 potential participants. One participant withdrew consent; one turned out to not be legally competent. Seven participants were excluded from the final analysis due to the diagnosis of active malignancy, three due to increased ferritin or hemoglobin values and three due to incomplete data.

In the final cohort of 97 study participants, 68 (70%) were female (Table 2). Patients with anemia (*n* = 70) were further classified according to the criteria described in the Section 2; participants without anemia (*n* = 27) made up the control group. More than half of the anemia patients had IDA (*n* = 39, 58%), while six (19%) patients had AI and seven (10%) IDA + AI (Table 3). About a quarter of the patients (*n* = 18, 26%) were not unequivocally classified (UA). Characteristics of the whole cohort and anemia patients are presented in Table 2 and Table 3, respectively. Six participants (22%) in the control group had ferritin levels below the lower normal limit of reference value (<13 μg/L for women and 30 μg/L for men), i.e., having latent iron deficiency. Median level of vitamin B12 (vitB12) did not differ in anemia patients compared to controls, but varied among subgroup analyses, with two participants in the controls and four patients with anemia having a vitB12 concentration below the lower reference limit (<188 pmol/L). No patients with a vegan or vegetarian diet were involved in the study.

### 3.2. Univariate Analysis

A total of 617 metabolites and 227 calculated parameters were included in the study.

Based on the observed differences between groups and biological plausibility, the linear model used to compare patients with anemia and controls was adjusted for age and sex. In subgroup analyses, the model was additionally adjusted for hemoglobin and creatinine concentration. The addition of other potential covariates, such as the interval between food intake and sample collection, the presence of chronic disease, and medication use, did not meaningfully improve model fit (ΔAIC −0.77, −1.4, −0.56, respectively).

In the whole cohort, six metabolites and six calculated parameters had significantly different serum concentrations in patients with anemia compared to controls with FDR-corrected *p*-values < 0.05 (Table 4). Patients with anemia had decreased levels of three triglycerides (TGs) (TG(17:1_38:6), (TG(17:1_38:5), (TG(14:0_40:5)) and two phosphatidylcholines (PCs) (PC aa C38:4, PC ae C36:5), while the concentration of one triglyceride (17:1_36_3) was increased. An additional 49 metabolites showed a trend towards differing between anemia patients and controls (uncorrected *p*-value < 0.05), but did not remain significant after correction for multiple testing (Supplementary Table S1A). The highest proportion of altered concentrations was observed in bile acids, amino acids, and phosphatidylcholines (PC-s).

Patients with anemia had higher asparagine/aspartate and docosahexaenoic acid (DHA)/eicosapentaenoic acid (EPA) ratios, while the ratio of different PC-s to choline was decreased (Table 4). An additional 16 calculated parameters showed a trend towards differences between anemia patients and controls (uncorrected *p*-value < 0.05) but did not remain significant after correction for multiple testing (Supplemental Table S1B).

Two single metabolites (spermine, lysophosphatidylcholine (lysoPC) 20:4) and two calculated parameters (sum of polyamines, ratio of polyamines to ornithine) had statistically significant alterations in anemia subgroups (Table 5). The concentration of lysoPC 20:4 was highest among the patients with AI, while the concentration of spermine was highest in patients with IDA + AI. An additional 18 metabolites and 14 calculated parameters indicated potential differences (uncorrected *p*-value < 0.05) but did not remain significant after multiple testing (Supplemental Table S1A,B). Three calculated parameters showed potential to separate IDA from AI, with the symmetric dimethyl arginine (SDMA)/arginine (Arg) and ornithine (Orn)/Arg ratio being lower and citrulline (Cit)/Orn being higher in patients with IDA compared to AI (uncorrected *p*-value, Tukey’s post hoc test < 0.05) (Supplementary Table S1B).

### 3.3. Multivariate Analysis

**Figure 1:**
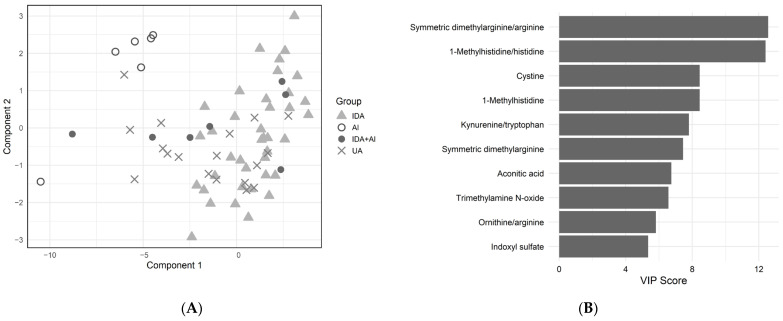
Multivariate analysis of metabolomics data in patients with iron deficiency anemia compared to anemia of inflammation. (**A**) sPLS-DA plot separating different anemia subtypes—IDA and AI used as training set; IDA + AI and UA test set. Model included 29 metabolites and calculated parameters—26 for calculating component 1 and 3 for component 2. (**B**) VIP scores of top 10 metabolites contributing to the separation in sPLS-DA model. IDA—iron deficiency anemia, AI—anemia of inflammation, UA—unclassifiable anemia, sPLS-DA—sparse partial least squares discriminant analysis.

In sPLS-DA, including only metabolomic data, keeping 29 metabolites or derived indices was found to be optimal for the stability of the model. The sPLS-DA model classified patients with a class-based error rate of 0.7% for IDA and 23% for AI (Figure 1A). The overall error rate of the model was 4%, and the balanced error rate was 12%, resulting in a general classification accuracy of 88% (95% confidence interval 83–93%). From the combined IDA + AI group, one sample was grouped together with AI, five with IDA and one in between with no clear dominance of IDA or AI profile. From the UA group, 11 grouped with IDA, one with AI, and six in between IDA and AI. SDMA/Arg was the variable with the highest VIP score, followed by 1-methylhistidine/histidine and cystine (Figure 1B).

## 4. Discussion

In this prospective cross-sectional study on metabolomic patterns related to anemia, the serum metabolome of anemic patients was first compared to controls independently of the cause of anemia. Thereafter, the metabolic profiles of anemias of different causes were examined, and finally, it was attempted to reclassify the UA group to IDA or AI based on their metabolite profile.

Differences in anemia and controls were most noted in triglycerides and PCs containing polyunsaturated fatty acids (PUFA).

The red blood membrane contains cholesterol and hundreds of different phospholipid molecules, including choline-containing PCs and sphingomyelins [26]. Enhanced phosphocholine metabolism has been shown to be essential for terminal erythropoiesis and oxidative phosphorylation, leading to higher levels of choline during terminal differentiation [27]. In AIHA patients, PC ae 34:3/PC ae 40:2 ratio was a predictor of AIHA relapse [13]. We observed that some PCs are significantly lowered in anemia but are inefficient in distinguishing anemia subtypes. Arachidonic acid-containing lysoPC C20:4 is an exception, probably due to higher demand for arachidonic acid in anemia and, particularly, in IDA + AI anemia. Triglycerides with polyunsaturated fatty acid (PUFA) residues were affected by anemia presence but not by anemia subtype.

One notable difference in patients with anemia compared to controls was an increased DHA/EPA ratio compared to controls. DHA and EPA are both omega-3 PUFAs; DHA can be synthesized from EPA in the human body. Although one step requires molecular oxygen and an iron-containing cytochrome delta-6-desaturase [28], this process is not hindered in anemia according to our results. In animal studies, hypoxia has been associated with an altered lipid profile in general and upregulated delta-6-desaturase at the mRNA level [29]. This may enhance EPA conversion to DHA and explain the decrease in EPA level and rise in DHA/EPA ratio also in our study.

Iron itself has a complex relationship with PUFAs. On one hand, iron deficiency has been shown to impair long-chain PUFA synthesis both in rats and humans [28]. Several studies have shown potential positive effects with additional PUFA supplements in iron supplements [30], including increased bioavailable iron and improved EPO resistance [31,32]. On the other hand, ferroptosis, a process of cell death by iron-induced lipid peroxidation, may be theoretically enhanced by co-supplementation [33]. Our study indicates reduced free and esterified PUFA levels in anemia. Further studies are needed to specify the cause of observed changes and investigate the potential role of EPA supplementation in the treatment of anemia.

We also observed a decrease in the AABA/Thr ratio in patients with anemia, in parallel with an increase in Thr with borderline significance. Thr has been associated with hypoxia, which could explain its increase in cases of anemia [34].

Cit and Cit/Orn ratio demonstrated borderline significance in comparisons between patients with anemia and controls. The Orn/Arg ratio emerged as a potential discriminator of IDA and AI in sPLS-DA and demonstrated borderline significance in univariate analysis together with Cit/Orn.

SDMA or SDMA/Arg emerged as a potential marker in discriminating IDA from AI in sPLS-DA, with borderline significance in univariate analysis. SDMA has been known to be elevated in chronic kidney diseases [35] and in vitro studies have shown that SDMA induces interleukin-6, tumor necrosis factor alpha expression [36], and reactive oxygen species production [37]. Contrary to that of asymmetric dimethyl arginine, the role of SDMA in nitric oxide metabolism is not so well established [38,39]. Our present results are in good accordance with the potential role of SDMA as a pro-inflammatory molecule. However, the exploratory nature of the cross-sectional study does not allow us to investigate causality, and the exact mechanism remains to be determined in further studies.

In our results of univariate analysis, spermine levels significantly differed among anemia subgroups, with the highest levels observed in patients with IDA + AI. Polyamines including spermine, spermidine and putrescine have been formerly described as inhibitors of erythropoiesis both in vitro [40] and in vivo [41]. Cellular iron has been shown to regulate polyamine synthesis [42]. Our results suggest that IDA and AI may have a cumulative enhancing effect on polyamine synthesis.

Alterations in different Arg metabolites have been previously described in other anemia subtypes. Homo-L-arginine level is decreased in Diamond-Blackfan anemia patients [18], N-acetylcitrulline level is increased in HS [16] and PKD [17]. Increased levels of different polyamines have been observed in PKD [17], HS [16], and AIHA patients with hemolytic activity [13]. Increased levels of ADMA have been observed in AIHA patients [13]. Whether all the alterations in arginine metabolites observed in our study stem from one root cause or are brought about by different circumstances remains unknown. Perturbances in nitrogen management and the urea cycle could explain some of the abovementioned changes, as well as the increased Asn/Asp ratio that we observed in anemia patients. However, SDMA and polyamines originate from minor pathways with specific regulatory roles rather than an expected outcome from a general metabolic imbalance.

The kynurenine-to-tryptophan (Trp) ratio, illustrating the activity of indoleamine 2,3-dioxygenase, separated AI and IDA in the sPLS-DA model. Indoleamine 2,3-dioxygenase is a heme enzyme, induced by proinflammatory cytokines, which converts tryptophan to kynurenine. Kynurenine is a well-established signal in chronic inflammation [43], being able to decrease EPO and increase hepcidin production in vitro [44]. Lower Trp and kynurenic acid concentrations in ID and increased kynurenine/Trp ratio in AI patients have been previously described [2,45]. We hypothesize that the opposing effects of iron deficiency and inflammation on the kynurenine/Trp ratio may enhance its ability to discriminate the two conditions.

For anemia subtype separation in the sPLS-DA model, our results included also indoxyl sulfate and trimethylamine N-oxide (TMAO). Indoxyl sulfate increases with renal disease and endothelial dysfunction [46], contributing to the genesis of anemia in chronic kidney disease, possibly via hepcidin production and eryptosis [47]. TMAO, a metabolite derived from gut microbiota and food, causes cardiovascular events and insulin resistance [47,48]. Further studies are needed to determine whether changes in the gut microbiome, rather than renal dysfunction itself, contribute to the elevated indoxyl sulfate-to-indole ratio and increased TMAO levels observed in AI in our dataset.

While the results of adjusted univariate analysis may detect the biomarkers with the highest potential for differential diagnosis, the sPLS-DA model offers a wider overview of metabolomic alterations. Classification of IDA + AI and UA groups based on the metabolomics sPLS-DA model indicated that most of these samples have more similarities with IDA than with AI. Some samples located between AI and IDA thus have characteristics from both conditions. However, the small number of patients in the AI subgroup resulted in a high class-based error rate, leading also to modest general classification accuracy (88%). In addition, the classification model has not been validated in an independent cohort, and validation of reclassification in the clinical setting (for example, response to iron therapy) has not been investigated. Furthermore, covariates not included in the sPLS-DA model may be the underlying factors responsible for the observed differences. Therefore, the results of multivariate analysis should be considered tentative and interpreted with caution.

This study has several limitations. The targeted metabolomic approach may lead to overlooking important alterations in pathways with metabolites not sufficiently represented in the commercial kit. Considering the dominance of IDA in patients with anemia, it is difficult to distinguish the effects of reduced Hb and ID. Furthermore, some participants in the control group had latent ID, possibly leading to less pronounced variation between the two groups.

The modest sample size and especially the uneven distribution of the subgroups limit the statistical power of the study. Moreover, the observed alterations in the metabolomic profile may have been driven by specific comorbid conditions rather than being universally associated with AI, thereby limiting the generalizability of the findings.

The anemia subgroups differed in age and renal function. Despite the use of adjustment for age, sex, renal function and hemoglobin in the subgroup comparison model, the potential influence of these factors on the observed alterations cannot be fully excluded. Furthermore, metabolomic profiles are known to be affected by recent food intake. Although inclusion of fasting status did not significantly alter the model, the potential impact of specific dietary exposures on metabolomic profiles cannot be ruled out. Due to the lack of bone marrow iron store assessment and universally accepted diagnostic criteria for AI, misclassification of participants into the respective subgroups cannot be completely ruled out. Due to the cross-sectional study design, the interpretations of the observed associations are hypothetical, and the precise mechanisms need to be elucidated in future studies.

As a strength, the samples were collected in the routine clinical setting, without dietary restrictions and prior fasting, potentially increasing the applicability to everyday practice. Moreover, not restricting the control group to only healthy participants should emphasize the robustness of detected alterations. Studies with larger datasets may be helpful in the search for physiological threshold values for Hb and ferritin, as previously studied with hepcidin [49]. Further research is needed to validate the results in larger cohorts and to test the potential causal relationships of observed alterations.

## 5. Conclusions

This study describes the metabolic changes connected to anemia, highlighting the alterations in PUFA metabolism in anemia. It also provides insight into the possible differentiating metabolomic patterns in IDA compared to AI, including modified arginine metabolism in AI. Our preliminary findings suggest that metabolomic data may be capable of distinguishing between IDA and AI. However, owing to several limitations of the current study, the potential utility of metabolome-based approaches should be confirmed in independent validation studies.

## Figures and Tables

**Table 1:** Classification of study subjects.

Group	Criteria
IDA	anemia no inflammation * no renal insufficiency ^†^ TSAT < 20% and ferritin < 30 μg/L OR sTfR-F index >= ≥ 3.7 in women and sTfR-F index ≥ 3.4 in men
AI	anemia inflammation and/or renal insufficiency TSAT < 20% and ferritin > 100 μg/L AND/OR sTfR-F index < 3.7 in women and sTfR-F index < 3.4 in men, and ferritin > 30 μg/L
IDA + AI	anemia inflammation and/or renal insufficiency TSAT < 20% and ferritin < 100 μg/L AND/OR sTfR-F index ≥ 3.7 in women and sTfR-F index ≥ 3.4 in men
UA	anemia did not fulfill any of the aforementioned criteria or fulfill the criteria of more than one category
Controls	no anemia no hyperferritinemia (women–ferritin up to 150 μg/L, men–ferritin up to 400 μg/L)

Classification is based on the criteria previously used by Santen et al., 2011 [20], including eGFR and modifying C-reactive protein and sTfR-F index cut-offs. IDA—iron deficiency anemia, AI—anemia of inflammation, UA—unclassifiable anemia, TSAT—transferrin saturation, sTfR-F—soluble transferrin receptor/log_10_(ferritin). * Active inflammation–C-reactive protein > 5 mg/L, ^†^ renal insufficiency–eGFR < 60 L/min/1.73 m^2^.

**Table 2:** Overview of the patients with anemia compared to the control individuals.

	Patients with Anemia	Controls	*p* Value
Count, *n*	70	27	
Fasting samples, *n* (%)	17 (24%)	10 (37%)	0.480
Women, *n* (%)	50 (71%)	18 (67%)	0.832
Age, years	49 (36–70)	41 (33–55)	0.048 *
BMI, kg/m^2^	26 (22–30)	25 (21–28)	0.536
Hb, g/L	105 (93–116)	139 (129–145)	<0.001 *
MCH, pg	26 (22–29)	29 (28–31)	<0.001 *
MCV, fL	83 (75–91)	87 (85–91)	0.01 *
Ret, ×10^9^/L	55 (44–66)	59 (51–81)	0.269
RET-He, pg	28 (23–33)	34 (32–35)	<0.001 *
WBC, ×10^9^/L	6 (5–7)	6 (5–7)	0.841
Plt, ×10^9^/L	275 (220–332)	251 (197–293)	0.083
Ferritin, µg/L	16 (8–50)	35 (19–90)	0.006 *
TSAT, %	9 (6–21)	23 (14–32)	<0.001 *
sTfR, mg/L	6 (4–8)	4 (3–5)	0.018 *
EPO, U/L	37 (23–64)	13 (9–17)	<0.001 *
CRP, mg/L	1 (1–2)	1 (1–2)	0.185
Creatinine, µmol/L	67 (60–83)	64 (58–74)	0.414
Folate, nmol/L	18 (12–30)	22 (15–36)	0.117
VitB12, pmol/L	362 (270–462)	353 (299–427)	0.932

Data is presented as median (interquartile range), unless otherwise specified. *p*-value is calculated with the Chi-squared test for sex and fasting samples, and with the Wilcoxon rank-sum test for all other variables. * *p* <0.05, statistically significant difference. BMI—body mass index, Hb—hemoglobin, MCH—mean corpuscular hemoglobin, MCV—mean corpuscular volume, Ret—reticulocyte count, RET-He—reticulocyte hemoglobin, WBC—white blood cell count, Plt—platelet count, TSAT—transferrin saturation, sTfR—soluble transferrin receptor, EPO—erythropoietin, CRP—C-reactive protein, vitB12—vitamin B12.

**Table 3:** Overview of the patients with different anemia subtypes.

	IDA	AI	IDA + AI	UA	*p* Value
Count, *n* (%)	39 (56%)	6 (9%)	7 (10%)	18 (26%)	
Fasting samples, *n* (%)	11 (28%)	1 (17%)	3 (43%)	4 (22%)	0.685
Women, *n* (%)	31 (80%)	2 (33%)	5 (71%)	12 (67%)	0.217
Age, years	43 (34–60)	76 (70–77)	73 (41–80)	59 (46–66)	0.007 *
BMI, kg/m^2^	26 (23–28)	25 (21–30)	29 (26–30)	25 (21–29)	0.724
Hb, g/L	102 (93–111)	101 (98–112)	88 (73–94)	117 (114–119)	<0.001 *
MCH, pg	25 (21–26)	30 (28–31)	22 (19–26)	30 (28–31)	<0.001 *
MCV, fL	79 (73–84)	94 (85–95)	76 (68–84)	91 (85–95)	<0.001 *
Ret, ×10^9^/L	55 (44–67)	60 (54–84)	65 (50–68)	48 (38–61)	0.251
RET-He, pg	25 (23–29)	34 (32–35)	24 (20–27)	34 (32–35)	<0.001 *
WBC, ×10^9^/L	5 (5–7)	6 (4–8)	7 (7–8)	7 (6–8)	0.068
Plt, ×10^9^/L	295 (231–347)	224 (199–268)	293 (246–325)	256 (223–280)	0.109
Ferritin, μg/L	8 (7–12)	605 (363–663)	15 (9–18)	56 (39–103)	<0.001 *
TSAT, %	7 (4–8)	28 (21–36)	9 (7–13)	23 (19–28)	<0.001 *
sTfR, mg/L	7 (6–9)	3 (2–5)	8 (6–9)	3 (2–4)	<0.001 *
EPO, U/L	43 (29–69)	38 (22–49)	76 (43–162)	14 (10–42)	0.002 *
CRP, mg/L	1 (1–1)	2 (1–6)	5 (5–6)	1 (1–2)	<0.001 *
Creatinine, µmol/L	63 (59–73)	111 (88–125)	76 (60–136)	69 (63–82)	0.012 *
Folate, nmol/L	19 (12–33)	20 (15–34)	9 (7–18)	15 (13–20)	0.298
VitB12, pmol/L	386 (325–468)	222 (201–313)	259 (214–314)	353 (314–474)	0.012 *

Data are presented as median (interquartile range), unless otherwise specified. *p*-value is calculated with a Chi-squared test for sex and fasting samples and with a Kruskal–Wallis test for all other variables. * *p* <0.05, statistically significant difference. IDA—iron deficiency anemia, AI—anemia of inflammation, UA—unclassifiable anemia, BMI—body mass index, Hb—hemoglobin, MCH—mean corpuscular hemoglobin, MCV—mean corpuscular volume, Ret—reticulocyte count, RET-He—reticulocyte hemoglobin, WBC—white blood cell count, Plt—platelet count, TSAT—transferrin saturation, sTfR—soluble transferrin receptor, EPO—erythropoietin, CRP—C-reactive protein, vitB12—vitamin B12.

**Table 4:** Serum concentration of metabolites and calculated parameters that differentiated between patients with anemia and controls.

	Patients with Anemia	Controls	FDR Corrected *p* Value	Fold Change	Cohen’s d
Single metabolites					
TG (17:1_38:6)	0.097 (0.021–0.27)	0.27 (0.22–0.37)	<0.001 *	0.36	−0.71
PC aa C38:4	63 (53–79)	78 (65–94)	0.011 *	0.81	−0.69
PC ae C36:5	8.0 (6.4–9.9)	10 (8.6–12)	0.015 *	0.78	−0.73
TG(17:1_38:5)	0.17 (0.011–0.41)	0.40 (0.31–0.49)	0.015 *	0.42	−0.66
TG(14:0_40:5)	1.1 (0.88–1.9)	2.1 (1.4–2.8)	0.022 *	0.52	−0.70
TG(17:1_36:3)	1.4 (0.90–1.9)	0.81 (0.57–1.4)	0.031 *	1.71	0.63
Calculated parameters					
Asparagine/aspartate	2.4 (1.8–3.0)	1.7 (1.5–2.4)	0.022 *	1.42	0.62
PC (O-)xx:x/choline	10 (8.1–14)	13 (11–16)	0.022 *	0.80	−0.66
AABA/threonine	0.11 (0.089–0.14)	0.15 (0.12–0.18)	0.032 *	0.73	−0.76
PC xx:x/choline	105 (79–132)	116 (108–159)	0.034 *	0.91	−0.60
PC O-xx:x/choline	94 (71–117)	103 (96–143)	0.039 *	0.91	−0.59
DHA/EPA	14 (3.1–21)	3.1 (2.1–15)	0.043 *	4.52	0.68

Data are presented as median (interquartile range), single metabolite concentrations in μmol/L, and calculated parameters in (μmol/L)/(μmol/L). * *p* < 0.05, statistically significant difference FDR—false discovery rate correction by Benjamini–Hochberg procedure. TG—triglyceride, PC aa—diacyl phosphatidylcholine, PC ae—acylalkyl phosphatidylcholine, PC (O-)xx—all PC-s, AABA—α-aminobutyric acid, PC xx—diacyl PC-s, PC O-xx—alkylacyl PC-s, DHA—docosahexaenoic acid, EPA—eicosapentaenoic acid.

**Table 5:** Serum concentration of metabolites and calculated parameter differences between anemia subgroups.

	IDA	AI	IDA + AI	UA	FDR Corrected *p* Value
Single metabolites					
Spermine	0.085 (0.070–0.16)	0.083 (0.076–0.12)	0.16 (0.12–0.22)	0.072 (0.069–0.093)	0.008 *^, §, ¶^
LysoPC C20:4	4.9 (4.1–6.2)	5.6 (4.3–6.7)	3.3 (3.2–3.7)	5.4 (4.7–6.3)	0.036 *^, §, ¶^
Calculated parameters					
Sum of polyamines	0.37 (0.33–0.46)	0.32 (0.28–0.38)	0.45 (0.38–0.47)	0.31 (0.26–0.40)	0.005 *^, §, ¶^
Sum of polyamines/ornithine	0.0060 (0.0043–0.0092)	0.0029 (0.0020–0.0039)	0.011 (0.0065–0.014)	0.0037 (0.0025–0.0061)	0.009 *^, §, ¶^

Data are presented as median (interquartile range), single metabolite concentrations in units μmol/L, and calculated parameters in (μmol/L)/(μmol/L)). FDR—false discovery rate correction by Benjamini–Hochberg procedure. IDA—iron deficiency anemia, AI—anemia of inflammation, UA—unclassifiable anemia, lysoPC—lysophosphatidylcholine, sum of polyamines—putrescine + spermidine + spermine. Post hoc test groupwise comparisons with uncorrected *p* < 0.05: * IDA vs. IDA + AI, ^§^ AI vs. IDA + AI, ^¶^ IDA + AI vs. UA.

## Data Availability

Due to ethical restrictions related to participant privacy and the conditions of the Research Ethics Committee approval, the raw data underlying this study cannot be publicly shared. Pseudonymized data are available on request from the corresponding author in justified cases.

## Supplementary Materials

The following supporting information can be downloaded at: https://www.mdpi.com/article/10.3390/metabo16070498/s1, Supplementary Table S1A—the list of metabolites measured with the MxP 500 Kit that were included in the statistical analyses, their median values (µM) with interquartile range and corresponding statistical significances, Supplementary Table S1B—the list of calculated parameters included in the statistical analyses, along with their median values (µM/µM) with interquartile range and corresponding statistical significances.

## Abbreviations

The following abbreviations are used in this manuscript:

IDA	Iron deficiency anemia
AI	Anemia of inflammation
ID	Iron deficiency
AIHA	Autoimmune hemolytic anemia
HS	Hereditary spherocytosis
PKD	Pyruvate kinase deficiency
EPO	Erythropoietin
Hb	Hemoglobin
UA	Unclassifiable anemia
ANOVA	Analysis of variance
sPLS-DA	Sparse partial least squares discriminant analysis
VIP	Variable importance in projections
vitB12	Vitamin B12
PC	Phosphatidylcholine
ADMA	Asymmetric dimethyl arginine
SDMA	Symmetric dimethyl arginine
EPA	Eicosapentaenoic acid
lysoPC	Lysophosphatidylcholine
Asn	Asparagine
Asp	Aspartate
DHA	Docosahexaenoic acid
Cit	Citrulline
Orn	Ornithine
AABA	α-aminobutyric acid
Thr	Threonine
PUFA	Polyunsaturated fatty acid
Arg	Arginine
Cys	Cysteine
TMAO	Trimethylamine N-oxide
